# Objective: biochemical function

**DOI:** 10.3389/fgene.2014.00210

**Published:** 2014-07-08

**Authors:** Brian P. Anton, Simon Kasif, Richard J. Roberts, Martin Steffen

**Affiliations:** ^1^New England BiolabsIpswich, MA, USA; ^2^Bioinformatics Program, Boston UniversityBoston, MA, USA; ^3^Department of Biomedical Engineering, Boston UniversityBoston, MA, USA; ^4^Department of Pathology and Laboratory Medicine, Boston University School of MedicineBoston, MA, USA

**Keywords:** experimental validation, hypothetical proteins, crowdsourcing, high-throughput, traceability

DNA sequencing enables the discovery of new genes in high-throughput, low-cost experiments. Conversely, gene function is determined by low-throughput, high-cost experiments. This inverse relationship for these two types of data is a major impediment in meeting one of the major scientific challenges of our time—the understanding of genomes.

This mismatch in throughput is illustrated by considering the progress made for one of the earliest sequenced genomes, that of *Mycobacterium tuberculosis* H37Rv (*Mtb*). When its genome was published in 1998, more than a quarter of its genes had no known function (Cole et al., 1998). Our lack of knowledge about these approximately 1000 “conserved hypothetical” genes in *Mtb* represents a serious deficiency in our understanding of its biology. Now, after more than a decade of progress, our knowledge of those proteins' functions is essentially unchanged—there are still greater than 900 genes with no known function (Lew et al., 2011). In contrast, during this same period, the scientific community has sequenced approximately 18,000 new genomes (Pagani et al., 2012), containing millions of new hypothetical proteins. Apparently, the vector of our progress has tipped decisively away from data interpretation and comprehension, and toward mere data collection.

To address the issue of gene function testing and annotation for all microbes, we founded COMBREX (COMputational BRidge to EXperiments), an endeavor aimed at accelerating the rate of gene function validation (Anton et al., 2013). Two of COMBREX's more prominent initiatives were the creation of a comprehensive database for protein function data (http://combrex.bu.edu), and the deployment of a crowdsourcing platform to catalyze protein function experimentation. In the course of these two efforts, it became apparent that fundamental changes in approaches to the problem of protein function determination were needed if there was any hope of keeping pace with DNA sequencing. We suggest that the community work together to (1) re-establish the connection between existing gene annotation and the foundational experimental data that supports all annotation, (2) develop experiment design principles to help guide the identification of maximally informative targets for function validation, (3) invest in the development of higher-throughput approaches for the testing of protein function, and (4) provide an expedited publication pathway for reporting experimental results of gene function, analogous to the reporting of newly sequenced genomes in the journal “Standards in Genomic Sciences.”

## Comprehensive assessment of protein function status

We recently examined protein function status from greater than 1000 completely sequenced microbes (Anton et al., 2013). For 3.3 million identified genes, we can currently document experimentally determined functions for just 0.4% of the proteins (13,665 proteins). 76% of the proteins are annotated using computational methods, and the remaining 24% of proteins (close to 1 million) have no known, or predicted, functions. Thus, a very small number of experimental data points provides the foundation for an enormously disproportionate number of predicted gene function annotations. (While the total number of experimentally characterized proteins is unknown, we estimate the number to be above 50,000).

An unavoidable consequence of the fact that only a small proportion of annotations are based directly on experiment is that predicted functions are often based on weak chains of inference. This can greatly contribute to the proliferation of incorrect annotations. When a newly-discovered gene is annotated based on similarity to a experimentally characterized gene, it then, itself, becomes a source for future annotation. As a result, genes that will be annotated in the future may be annotated based upon genes that are themselves far removed from solid experimental evidence. Compounding confusion, in the vast majority of cases, the original experimental source has not been recorded or preserved. One study estimated that for 37 protein families and 7000 sequences, the overall misannotation rate is roughly 40% (Schnoes et al., 2009), yet the vast majority of annotations are frequently unquestioned by many working scientists.

## Crowdsourcing the experimental testing of protein function

In the first phase of the project, COMBREX awarded funds to 14 labs, and 140 proteins were examined. One of the primary criteria for these applications was prior published work using the proposed enzyme assay. The rationale for this was that experimental efficiency will be greatest, and the costs minimized, in laboratories that already have the reagents, equipment, and expertise necessary to perform the experiments quickly and accurately. Research on many of these proteins has been successfully completed, and results have been published (Chatterjee et al., 2012; Clark et al., 2012; Francis et al., 2012; Phillips et al., 2012; Rodionova et al., 2012; Su et al., 2012; Xu et al., 2012; Choi et al., 2013; Elkin et al., 2013), while research on the others is still in progress.

When a protein's function is experimentally determined, it not only affects its own annotation, it changes the probability that other proteins that are close in sequence space have a similar annotation. Thus the potential impact of the experiments COMBREX was able to fund is much larger than simply the proteins tested: the 140 proteins reside in Protein Clusters containing in total more than 3200 proteins, which are therefore quite close in sequence space and likely to have similar functions. At a further distance threshold, there are over 60,000 proteins that have BLAST *E*-values less than 1e-05. The 140 proteins have eight Pfam-defined domains of unknown function (DUFs), resulting in novel predictive insights for all other proteins containing these DUFs (a total of 1610 in the COMBREX Database). Finally, 37 of these 140 proteins contain a total of 28 unique Pfam-defined domains shared with human proteins, providing functional insights that may impact human health.

Several of the COMBREX awards went to labs that had participating undergraduate students, highlighting that the types of experiments COMBREX funds meshes well with the interests and capabilities of undergraduate students eager to participate in original research, and with STEM educational goals of many science departments. As an example, undergraduate students at the University of Virginia were able to successfully determine biochemical activities and enzyme kinetics for three uncharacterized proteins (Elkin et al., 2013). COMBREX hopes to replicate these successes as part of an educational component at numerous undergraduate institutions, in a manner analogous to the Small World Initiative, developed at Yale, which enlists undergraduates in the search for new antibiotics (Barral et al., 2014).

## Connecting annotation to experimental sources

When confronted today with the task of annotating a newly discovered hypothetical protein, the use of BLAST quickly and robustly identifies homologous proteins. This sometimes provides clues to potential gene function. However, just as often, one is inundated with matches to other hypothetical proteins that reveal little about possible gene function, and obscures similarities to experimentally characterized proteins.

We developed a prototype tool, named COMBLAST, to associate query genes with the various types of experimental evidence and data stored in COMBREX. COMBLAST returns results summarized in a format that concisely captures the functional features of similar proteins. COMBLAST output includes a trace to experimental evidence of function via sequence and domain similarity, to available structural information for related proteins, to association with clinically relevant phenotypes such as antibiotic resistance, and other relevant information.

The first application of COMBLAST was deployed in a collaboration led by D. Wood and S. Salzberg (Wood et al., 2012). We analyzed 1474 prokaryotic genome annotations in GenBank and identified 25,394 potential genes that were very likely overlooked during the original annotations. COMBREX was able to provide supporting evidence of their protein-coding nature, and we were able to associate 13,633 of the proteins to published biochemical evidence. Providing explicit links to documented proteins represents one approach for supporting annotations of “missing proteins” (Lane et al., 2014), until comprehensive proteomic surveys confirm their expression (Kim et al., 2014). While an efficient and user-friendly interface to the COMBLAST software is under development, when finally deployed, it will enable any scientist to quickly re-assess the validity of any existing annotation, or to generate hypotheses based solidly on existing experimental evidence.

## Designing experiments with increased information content

The ability to only perform a small number of experiments places a premium on every attempted experiment, making an important consideration the possible amount of information that will be derived from any one experiment. This “information gain” from the experimental analysis of a given protein is dependent on the number of proteins nearby to it in sequence space, as well as the distances of that protein to previously characterized proteins.

In the most simplistic sense, characterization of a judiciously chosen protein generates or improves predictions for many other proteins across many genomes, while characterization of a protein related to few or no other proteins may have a much smaller impact. More formally, for function prediction methods that report probabilities with their predictions (Letovsky and Kasif, 2003), the information gain from an experiment can be quantified as the reduction in the estimated probability of prediction error, summed across all predictions.

In COMBREX, we implemented a proof-of-concept prioritization scheme that ranked proteins for experimental testing, which roughly paralleled expected trends in information gain. The “ideal” COMBREX target is a protein close to many other uncharacterized proteins, and relatively far from any protein of known function, but not so far that it would preclude high quality predictions of the protein's function for the experimentalist to test. A second, “soft” guideline was the encouragement to test more than a single protein within a family. Typically, there is only a marginal increase in labor to biochemically test three similar proteins in parallel, when one has procured all the reagents, and created all the buffers for the testing of a single protein, yet the information gain can be significantly increased, as one starts to define boundaries of spaces in which contain proteins with a specific function. Put another way, these design principles do not provide answers—they help experimentalists ask better questions.

## Development of high-throughput technologies for gene function determination

The functional characterization of hypothetical proteins with only remote sequence homology to known proteins can be challenging, as there may be few clues to guide initial experiments. Several groundbreaking efforts have circumvented this obstacle by deploying technologies that utilize a large diverse set of reagents, or cast their net over a large, complex pool of proteins. Yakunin and coworkers (Kuznetsova et al., 2005; Proudfoot et al., 2008) screen individual proteins for general activity using a set of reagents selected to be generically active (testing for broad functionalities, such as phosphatase, dehydrogenase, protease, etc.), which is then followed by the use of more specific substrates. Cravatt and coworkers (Cravatt et al., 2008; Simon and Cravatt, 2010) have pioneered a complementary approach, “activity-based protein profiling,” enriching enzymes of a particular class using reagents that contain affinity labels, reactive groups and a tag for isolation, and then identifying proteins by mass spectrometry. They and others have applied this technique to multiple classes of enzymes including: hydrolases, proteases, kinases, phosphatases, histone deacetylases, glycosidases, and oxidoreductases.

We have recently developed a workflow for the characterization of hypothetical proteins and applied it to six proteins from *H. pylori* (Choi et al., 2013). We utilized an affinity method to generate initial hypotheses for hypothetical proteins, and then confirmed reactivity using standard recombinant DNA technology and traditional *in vitro* biochemistry. The affinity reagents utilize nano-particles coated with substrate analogs to enrich proteins from cell lysates of *H. pylori*. Isolated proteins were identified using mass spectrometry. After cloning and expression in *E. coli*, the proteins were tested for biochemical activities related to the molecular fragment serving as the affinity bait. Proteins characterized include a guanosine triphosphate (GTP) cyclohydrolase (HP0959), an ATPase (HP1079), an adenosine deaminase (HP0267), a phosphodiesterase (HP1042), an aminopeptidase (HP1037), and new substrates were characterized for a peptidoglycan deacetylase (HP0310).

## The need for convenient publication pathways for improved dissemination of results

We suspect that a tremendous amount of pertinent experimental gene function information is lost to the community at large because of difficulties associated with finding appropriate venues to disseminate the information. The genomics community addressed this need smartly with the creation of an open access journal, *Standards in Genomic Sciences*. This journal typically publishes short, straightforward descriptions reporting a new genome sequence based on a standard template.

There is a need for a similar publication mechanism for gene function data. It appears that currently, the scientific community's publication standards generally dictate that a successful biochemical experiment alone does not meet the criteria for a minimum publishable unit. Without accompanying data about the gene's role in the biology of the organism, or observations on associated phenotypic effects, biochemical results are not “enough” of a story. As a result, useful experimental information remains hidden in individual notebooks, lost to the wider community.

In our opinion, there would be great value in a publication venue that accepted streamlined “biochemical reports” in a routine manner. Minimal data provided would be the sequence of the gene, the protein production method, the biochemical assay, and an interpretation of the results. Similarly, simple reports on gene overexpression or knockouts and their phenotypic effects would permit the dissemination of meaningful functional data. Such data could be linked to COMBREX and other frequently accessed gene databases to expedite the dissemination process by avoiding human curation or processing.

## Summary

There needs to be a paradigm shift in the approach taken to determine and assign gene function if there is to be any hope of realizing the potential benefits from the torrent of new genome sequences. We advocate here for: (1) experimental designs that test sets of maximally informative proteins, (2) maximal information extraction from every experimental result, with explicit traces provided to related proteins, (3) enhanced opportunities for collaboration among computational and experimental researchers to share predictions and results, and distribute limited resources, (4) investment by granting agencies in the development of high-throughput gene function testing, and (5) the creation of new publication options to report and share the results of experiments that are performed.

## Funding

COMBREX is funded by a GO grant from the National Institute of General Medical Sciences (NIGMS) (1RC2GM092602-01). The funders had no role in study design, data collection and analysis, decision to publish, or preparation of the manuscript.

### Conflict of interest statement

The authors declare that the research was conducted in the absence of any commercial or financial relationships that could be construed as a potential conflict of interest.
